# Trends and Determinants of Antiretroviral Therapy Patient Monitoring Practices in Kenya and Uganda

**DOI:** 10.1371/journal.pone.0135653

**Published:** 2015-08-14

**Authors:** Emily Dansereau, Emmanuela Gakidou, Marie Ng, Jane Achan, Roy Burstein, Brendan DeCenso, Anne Gasasira, Gloria Ikilezi, Caroline Kisia, Samuel H. Masters, Pamela Njuguna, Thomas A. Odeny, Emelda A. Okiro, D. Allen Roberts, Herbert C. Duber

**Affiliations:** 1 Institute for Health Metrics and Evaluation, University of Washington, Seattle, Washington, United States of America; 2 Department of Pediatrics and Child Health, Makerere University, Kampala, Uganda; 3 RTI International, Research Triangle Park, North Carolina, United States of America; 4 African Leaders Malaria Alliance, Kampala, Uganda; 5 Action Africa Health-International, Nairobi, Kenya; 6 University of North Carolina, Chapel Hill, North Carolina, United States of America; 7 IFC World Bank Group, Nairobi, Kenya; 8 Department of Epidemiology, University of Washington, Seattle, Washington, United States of America; 9 Bill & Melinda Gates Foundation, Seattle, Washington, United States of America; University of Missouri-Kansas City, UNITED STATES

## Abstract

**Introduction:**

Patients receiving antiretroviral therapy (ART) require routine monitoring to track response to treatment and assess for treatment failure. This study aims to identify gaps in monitoring practices in Kenya and Uganda.

**Methods:**

We conducted a systematic retrospective chart review of adults who initiated ART between 2007 and 2012. We assessed the availability of baseline measurements (CD4 count, weight, and WHO stage) and ongoing CD4 and weight monitoring according to national guidelines in place at the time. Mixed-effects logistic regression models were used to analyze facility and patient factors associated with meeting monitoring guidelines.

**Results:**

From 2007 to 2012, at least 88% of patients per year in Uganda had a recorded weight at initiation, while in Kenya there was a notable increase from 69% to 90%. Patients with a documented baseline CD4 count increased from 69% to about 80% in both countries. In 2012, 83% and 86% of established patients received the recommended quarterly weight monitoring in Kenya and Uganda, respectively, while semiannual CD4 monitoring was less common (49% in Kenya and 38% in Uganda). Initiating at a more advanced WHO stage was associated with a lower odds of baseline CD4 testing. On-site CD4 analysis capacity was associated with increased odds of CD4 testing at baseline and in the future.

**Discussion:**

Substantial gaps were noted in ongoing CD4 monitoring of patients on ART. Although guidelines have since changed, limited laboratory capacity is likely to remain a significant issue in monitoring patients on ART, with important implications for ensuring quality care.

## Introduction

Over the past decade there has been a massive scale-up of antiretroviral therapy (ART) in sub-Saharan Africa. Between 2003 and 2012, the number of people receiving ART in the region rose from 100,000 to 7.5 million [1,2]. Kenya and Uganda were among the countries that rapidly expanded ART services during this time. In Kenya, the number of patients receiving ART more than doubled between 2008 and 2013, rising from 237,881 to 656,359 [3]. In Uganda, the number of people on ART has increased by more than 100,000 per year, from 313,117 in 2011 to 570,486 by late 2013 [4,5]. With more patients on treatment, the need to evaluate patients for ART eligibility, and monitor patients once on treatment, has also increased.

At the time of ART initiation, proper evaluation can identify severely underweight patients, as well as those with very low CD4 counts who are at higher risk of poor outcomes and may require closer clinic and provider follow-up [6,7]. Further, clinical staging and/or CD4 counts are the primary eligibility criteria for ART initiation among non-pregnant adults in most of sub-Saharan Africa [8].

Once initiated, monitoring a patient’s response to therapy allows providers to detect potential adherence problems and treatment failure. It is critical to promptly identify treatment failure, as switching to a second-line regimen for these patients is essential for reducing mortality risk and preventing the spread of drug resistance [9–11]. Until recently, guidelines relied heavily on clinical and immunological monitoring, a combination that has been shown to be superior than clinical monitoring alone [12–16]. However, the World Health Organization (WHO), Kenya, and Uganda have recently recommended viral load monitoring, given its better sensitivity, specificity, and timeliness in detecting treatment failure [8,17–20]. Nonetheless, clinical and immunological measures are still recommended in the absence of viral load testing.

Despite the need for risk stratification and ongoing monitoring while on ART, relatively little is known about how these guidelines are followed in practice. Laboratory testing in sub-Saharan Africa has largely been hindered by inadequate infrastructure, equipment, and reagent shortages, and low availability of skilled laboratory professionals [21,22]. Additionally, monitoring requires regular care-seeking by patients with HIV, actions that can be limited by financial, geographical, and psychosocial factors [23–26].

As Kenya and Uganda pursue new monitoring guidelines that include regular viral load testing, it is important to understand how well prior guidelines were followed and to consider why they may have fallen short in practice. In this paper, we assess recent CD4 and weight monitoring practices and examine facility- and patient-level characteristics associated with meeting national monitoring guidelines.

## Methods

### Study sample

This study used data from a subset of facilities from a larger, multi-country facility-based project that took place in Kenya and Uganda (Access, Bottlenecks, Costs, and Equity [ABCE] project) [27]. Nationally representative facility samples were constructed for Kenya and Uganda using a two-step, stratified random sampling process detailed elsewhere [28,29]. In sum, subnational units (districts or counties) were stratified by country-specific characteristics (e.g., socioeconomic features, access to health care, etc.), and districts or counties were randomly selected from each stratum; urban epicenters (Kampala for Uganda; Nairobi and Mombasa for Kenya) were purposely included in addition to randomly selected districts or counties. Within each randomly selected district or county, facilities were stratified by their government-determined level of complexity and then randomly selected until a pre-determined quota was met for each facility type.

Health facilities that declined study participation or where access to the facility was limited due to safety, travel distance, or time constraints were replaced with other similar facilities within the same district by the country team when a suitable replacement facility was identified. Data collection took place from April to November 2012 in both countries.

At all selected facilities that provided ART services, we conducted a retrospective chart review of adult patients (18 years and older) who initiated ART 6 to 60 months prior to the survey date. In addition to patients actively in care, we sought to include the charts of all transferred, defaulted, and deceased patients. We received electronic medical records for all patients meeting inclusion criteria at four facilities in Kenya. At the remaining facilities in both countries, the facility administrator reported the total number of eligible charts, and we sampled up to 250 charts using an equal-probability procedure. At facilities with less than 250 charts, we included all charts in our study. Two facilities in Uganda provided electronic records for the sampled patients, while all other information was extracted from paper charts.

### Data collection

Trained research associates interviewed facility administrators to collect information about facility management, resources, practices, and patient volumes. They then extracted information related to patient demographics, initiation characteristics, treatment regimen, outcomes, full visit history, and all test results (CD4 count, weight, and viral load) from the sampled ART charts. Research associates also searched each patient’s folder for documentation of weight, CD4 count, or viral load tests that were not recorded in the standard chart and extracted the relevant information.

We applied sample weights based on the reported number of adult patients in each facility’s ART program, such that our reported values are representative of all patients at the sampled facilities. Analyzes related to initiation were weighted based on the annual number of new initiates, while analyzes for ongoing monitoring used the annual number of enrolled patients. We linearly extrapolated patient numbers for the 9% of facility-years where these data were not reported. We also extrapolated patient numbers for all facilities in 2012 as the facility survey only collected information through 2011.

### Describing monitoring practices in relation to minimum guidelines

We examined whether each patient met existing national guidelines for minimum monitoring at baseline and once they were established on therapy. These guidelines were consistent across countries and years for our study period (2007–2012) [15,16,30].

At ART initiation, both countries called for a measure of weight, CD4 cell count, and WHO stage. We determined whether each measurement was recorded at some time between three months prior to and one month following initiation to account for lags between eligibility testing and actual ART initiation; this approach also accounted for the potential that tests performed at ART initiation could be reviewed and recorded at a subsequent visit.

After the initiation period (typically defined as the first six months) monitoring guidelines called for a weight measurement every three months and CD4 measurement every six months, at minimum [15,16,30]. For each calendar month from 2009 to 2012, we restricted our denominator to patients who were (a) *established*, meaning they initiated ART at least six months before the given month, and (b) *retained*, meaning they were alive and on treatment during the given month, and not transferred, dead, or defaulted (i.e., no visit for six months and never returning). A patient met CD4 testing guidelines for a given month if a CD4 test was recorded at any point in the prior six months. Likewise, they met weight guidelines if a weight measure was recorded in the prior three months. If a patient did not have a test recorded during a specified time period, we determined whether the patient visited the facility during that period without receiving a test. Results for a given facility type-country-month are only shown if at least 50 charts were included in the calculation.

### Determinants of baseline and routine monitoring

We used a series of mixed-effects logistic regressions to assess factors associated with meeting minimum guidelines. For each country, models were run at the patient level using facility random effects.

Two separate analyzes were performed. The first examined determinants of having a baseline CD4 test and weight measurement, run as separate models, among patients who initiated therapy between 2011 and 2012. This group, the mostly recently initiating patients in our sample, was selected because determinants of testing may have changed over time and more recently measured factors are most relevant to policy and practice.

The second set of analyzes examined the extent to which established patients were meeting monitoring guidelines at the time of the survey. Patients were included in this analysis if (1) they were alive and in care at the time of the survey rather than transferred, dead, or defaulted; and (2) they had initiated at least 12 months prior to the survey, to ensure results were not influenced by elevated testing in the period immediately following initiation. Separate models examined two binary dependent variables: (1) CD4 test recorded during the six months prior to record extraction; and (2) weight recorded in the three months prior to record extraction.

Independent variables were selected *a priori* based on potential theoretical relationships with monitoring practices. All models included patient-level characteristics (age and sex), as well facility-level indicators including facility type (hospital or health center); ownership (public or private); age of ART program; on-site CD4 analysis capacity; receipt of HIV-specific staff training during the past year; and whether nurses led patient treatment.

Models assessing baseline CD4 and weight measurements included an indicator of baseline disease severity as categorized by four WHO stages.

Models examining recent measurements for established patients included duration on ART and binary indicators capturing whether the patient showed signs of WHO-defined immunological or clinical failure in the six months preceding the testing window of interest. Per WHO definitions [8], potential immunological failure was defined as having a CD4 count lower than 100 or experiencing a CD4 count falling to baselines levels. Potential clinical failure was defined as experiencing weight loss exceeding 10% from the patient’s highest weight. Other clinical criteria could not be included due to inconsistent recordkeeping.

All analyzes were conducted in Stata 13.1 (Stata Corp., College Station, Texas, USA).

### Ethical considerations

Ethical approval for this study was obtained from the University of Washington Human Subjects Division and local institutional review boards in Kenya (Kenya Medical Research Institute Ethics Review Committee) and Uganda (Makerere University School of Medicine Research Ethics Committee). Patient consent for review of clinical charts was not obtained, as all information extracted from clinical charts was anonymized and de-identified prior to analysis.

## Results

Data from 23,618 patient charts were extracted from 97 facilities offering ART (15,671 charts from 51 facilities in Kenya and 7,947 charts from 46 facilities in Uganda). Table 1 provides an overview of sampled facility and patient characteristics.

**Table 1:** Characteristics of sampled facilities and patients at initiation.

Indicator	Kenya	Uganda	Total
**Facility characteristics**			
Total number of facilities	51	46	97
Median program start year	2006	2005	2005
Level of complexity			
Hospital	53%	59%	56%
Health center	47%	41%	44%
Facility ownership			
Government or NGO	90%	67%	79%
Private	10%	33%	21%
Location			
Urban or peri-urban	71%	63%	67%
Rural	29%	37%	33%
Monitoring capacity			
Functional adult scale	100%	98%	99%
Functional measuring tape	94%	89%	92%
On-site CD4 analysis	29%	80%	54%
On-site viral load analysis	4%	2%	3%
Guidelines and training			
Has HIV guidelines	96%	98%	97%
Staff received HIV training in last year	16%	37%	26%
Has nurse-led HIV treatment	41%	28%	35%
**Patient characteristics**			
Total number of patients	15,671	7,947	23,618
Percent female	66%	61%	64%
Median age (years)	37	35	36
18–29	21%	29%	24%
30–39	38%	39%	39%
40–49	26%	22%	24%
50+	15%	10%	13%
Year of initiation			
2007	6%	5%	6%
2008	18%	11%	16%
2009	20%	15%	18%
2010	24%	22%	23%
2011	26%	31%	28%
2012	5%	16%	9%
WHO Stage			
Stage 1	23%	22%	23%
Stage 2	36%	38%	36%
Stage 3	37%	33%	36%
Stage 4	4%	7%	5%
Median baseline CD4 count	172	180	175

### Facility characteristics

The sampled facilities were largely publicly-owned (79%) and urban (67%). Most facilities had HIV guidelines available (97%), but only 26% had staff with HIV-specific training during the previous year. Ugandan facilities in our sample (80%) were more likely to have on-site CD4 analysis capacity than those in Kenya (29%). Most facilities without CD4 analysis capacity took samples on-site and shipped them elsewhere for analysis; only 3% did not offer any CD4 testing. One hospital each in Uganda and Kenya reported capacity to run viral load assays. Functional adult weight measurement scales (99%) and measuring tapes (92%) were nearly universally available.

### Patient characteristics

The majority of patients were female (64%) with a median age of 36 at initiation. When recorded, the median baseline CD4 count was 175 and 41% of patients were classified as WHO stage 3 or 4. Over half of patients initiated in 2010 or 2011.

### Baseline monitoring

**Fig 1:**
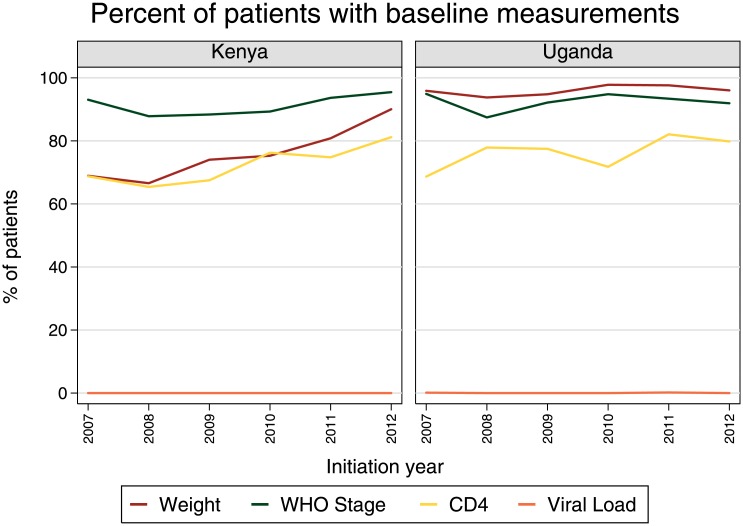
Percent of patients receiving baseline measurements by initiation year, 2007–2012.

Between 2007 and 2012, at least 88% of patients had a WHO stage recorded at initiation across country-years (Fig 1). Weight was recorded for over 94% of patients from all initiating cohorts in Uganda, while the percentage of Kenyan patients with a baseline weight measurement increased from 69% in 2007 to 90% in 2012. Baseline CD4 testing rates also increased between 2007 and 2012, rising from 69% to 80% in Uganda and from 69% to 81% in Kenya. Less than 1% of patients received a baseline viral load measure.

### Monitoring of established patients

**Fig 2:**
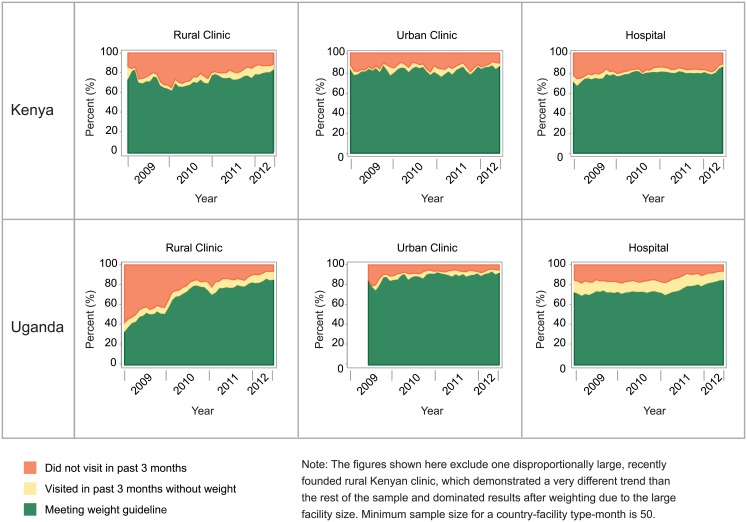
Percent of established patients meeting weight monitoring guidelines, 2009–2012.

In 2012, 83% and 86% of established patients met the recommended quarterly weight monitoring in Kenya and Uganda, respectively (Fig 2). This was an improvement from 2009, when 76% of Kenyan and 72% of Ugandan patients met weight-monitoring guidelines. Trends in weight monitoring were either stable or showed improvement across facility types in both countries. In particular, 85% of patients at rural health centers in Uganda met weight monitoring guidelines in 2012, compared to 49% in 2009. By 2012, all types of facilities in both countries had at least 80% of established ART patients receiving the recommended weight measures. These gains occurred in parallel with increases in ART visit frequency, with the proportion of patients with a facility visit every three months increasing from 81% in 2009 to 90% in 2012.

**Fig 3:**
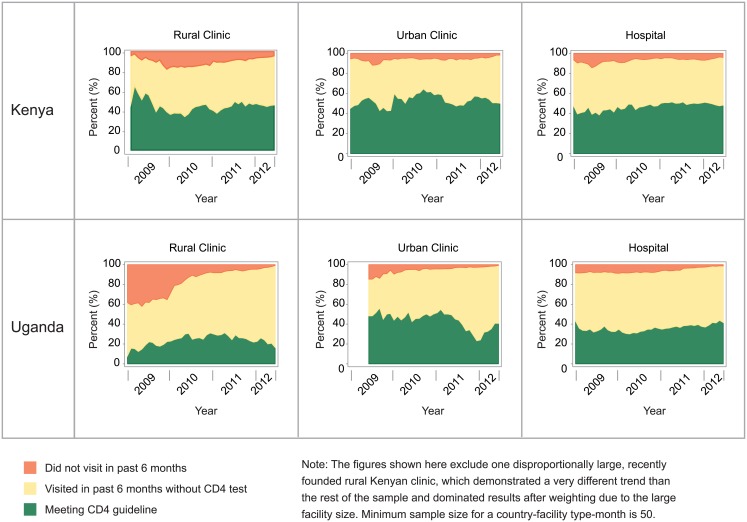
Percent of established patients meeting CD4 monitoring guidelines, 2009–2012.

In 2012, 49% and 38% of established ART patients met the guideline for semi-annual CD4 tests in Kenya and Uganda, respectively (Fig 3). This finding reflects minimal changes in CD4 testing rates since 2009 (43% in Kenya and 35% in Uganda). Rural health centers in Uganda had the lowest proportion of established patients who received semi-annual CD4 tests over time (22% in 2012). Kenyan hospitals showed the greatest improvement in CD4 testing rates, rising from 42% in 2009 to 49% in 2012. In contrast, urban health centers in Uganda experienced declines in CD4 testing during this time, falling from 50% to 35%. Notably, 97% of established ART patients had a clinical visit every six months in 2012, yet fewer than 50% of patients received a CD4 test every six months.

### Predictors of baseline testing

Controlling for patient and facility characteristics, a more advanced baseline WHO stage was associated with lower odds of baseline weight testing in Kenya (odds ratio [OR] = 0.49, 95% confidence interval [CI] = 0.30–0.80 for WHO stage 4 with respect to WHO stage 1) and lower odds of baseline CD4 testing in both countries (Kenya: OR = 0.56, 95% CI = 0.35–0.91; Uganda: OR = 0.53, 95% CI = 0.34–0.82) (Table 2).

**Table 2:** Predictors of receiving baseline measurements: mixed effects logistic regression results for patients initiating in 2011–12.

Weight at baseline	Kenya		Uganda	
*n*	4,806		3,002	
**Patient characteristics**				
Female	1.08	[0.91,1.28]	1.57*	[1.03,2.39]
Age at initiation	1.00	[1.00,1.01]	1.01	[0.99,1.03]
Initiation year	1.59**	[1.17,2.16]	0.51*	[0.30,0.88]
**Facility characteristics**				
Facility type (ref: Hospital)				
Urban health center	1.86	[0.73,4.71]	1.82	[0.50,6.71]
Rural health center	0.73	[0.33,1.61]	0.85	[0.25,2.98]
Government or NGO-owned	1.21	[0.36,4.08]	0.43	[0.16,1.16]
Staff HIV training in last year	1.12	[0.49,2.56]	0.83	[0.33,2.05]
Has nurse-led HIV treatment	1.04	[0.56,1.92]	2.06	[0.70,6.00]
Age of ART program	1.04	[0.87,1.26]	1.01	[0.80,1.28]
Has on-site CD4 analysis	1.31	[0.59,2.86]	1.36	[0.41,4.48]
**Clinical characteristics**				
WHO stage (ref: 1)				
Stage 2	0.79*	[0.62,0.99]	0.88	[0.47,1.65]
Stage 3	0.51***	[0.41,0.64]	0.95	[0.48,1.87]
Stage 4	0.49**	[0.30,0.80]	0.59	[0.22,1.56]
Not recorded	0.63*	[0.44,0.90]	0.09***	[0.05,0.17]
**CD4 at baseline**				
*n*	4,806		3,002	
**Patient characteristics**				
Female	0.98	[0.83,1.15]	1.14	[0.93,1.40]
Age at initiation	1.01**	[1.00,1.02]	1.01	[1.00,1.02]
Initiation year	1.45*	[1.07,1.97]	1.28	[0.96,1.71]
**Facility characteristics**				
Facility type (ref: Hospital)				
Urban health center	4.50**	[1.48,13.68]	0.57	[0.27,1.20]
Rural health center	0.42	[0.16,1.08]	0.22***	[0.10,0.46]
Government or NGO-owned	1.93	[0.44,8.47]	0.77	[0.43,1.36]
Staff HIV training in last year	4.10**	[1.50,11.26]	0.55*	[0.32,0.93]
Has nurse-led HIV treatment	0.78	[0.37,1.64]	2.47**	[1.34,4.54]
Age of ART program	0.89	[0.72,1.12]	0.92	[0.79,1.07]
Has on-site CD4 analysis	3.28*	[1.27,8.50]	2.63**	[1.31,5.28]
**Clinical characteristics**				
WHO stage (ref: 1)				
Stage 2	0.87	[0.70,1.08]	1.10	[0.83,1.45]
Stage 3	0.44***	[0.35,0.54]	0.71*	[0.53,0.95]
Stage 4	0.56*	[0.35,0.91]	0.53**	[0.34,0.82]
Not recorded	0.13***	[0.09,0.18]	0.23***	[0.16,0.34]

*p < 0.05

**p < 0.01

*** p < 0.001

Having on-site CD4 analysis capacity was associated with increased odds of baseline CD4 testing in Kenya (OR = 3.28, 95% CI = 1.27–8.50) and Uganda (OR = 2.63, 95% CI = 1.31–5.28). Compared to patients receiving care at hospitals, patients at urban health centers had increased odds of receiving a baseline CD4 test in Kenya (OR = 4.50, 95% CI = 01.48–13.68) and those at rural health centers had reduced odds in Uganda (OR = 0.22, 95% CI = 0.10–0.46). Kenyan patients at facilities with staff who received HIV training in the past year had an increased odds of a baseline CD4 test (OR = 4.10, 95% CI = 1.50–11.26); by contrast, receipt of HIV-specific training in Uganda was associated with a lower odds (OR = 0.55, 95% CI = 0.32–0.93). Ugandan patients at nurse-led programs had an increased odds for the receipt of baseline CD4 testing (OR = 2.47, 95% CI = 1.34–4.54).

### Predictors of established patients receiving recommended measurements during follow-up

In comparison with patients receiving ART at hospitals, Ugandan patients at urban health centers had an increased odds of meeting weight guidelines (OR = 3.42, 95% CI = 1.65–7.08) and those at rural health centers had a reduced odds of meeting CD4 guidelines (OR = 0.21, 95% CI = 0.08–0.53). Patients at urban health centers had increased odds of meeting CD4 testing guidelines in Kenya (OR = 2.15, 95% CI = 1.13–4.09).

Kenyan patients had an increased odds of receiving a CD4 test in the past six months if they received care at a facility with staff who received HIV-specific training during the last year (OR = 2.74, 95% CI = 1.50–5.02). In Uganda, such training was associated with an increased odds for patients meeting weight guidelines (OR = 1.74, CI = 1.05–2.89), but a reduced odds for meeting CD4 testing guidelines (OR = 0.49, 95% CI = 0.24–0.98). In Uganda, patients who received care at a HIV program with nurse-led care also had increased odds of meeting CD4 testing guidelines (OR = 2.49, 95% CI = 1.17–5.30).

For established patients in Uganda, prior indications of immunological failure were associated with an increased odds of meeting weight monitoring guidelines (OR = 2.60, 95% CI = 1.27–5.29), while a previous indication of clinical failure was associated with an increased odds of meeting the semi-annual CD4 testing guidelines (OR = 1.34, 95% CI = 1.00–1.80) (Table 3).

**Table 3:** Predictors of meeting CD4 and weight monitoring guidelines: mixed effects logistic regression results for established patients in care at the time of the survey.

Weight at time of survey	Kenya		Uganda	
*n*	5162		3867	
**Patient characteristics**				
Female	1.05	[0.87,1.27]	1.07	[0.89,1.28]
Age at time of survey	1.01**	[1.00,1.02]	1.00	[0.99,1.01]
Years on ART	1.15***	[1.07,1.24]	0.94	[0.87,1.02]
**Facility characteristics**				
Facility type (ref: Hospital)				
Urban health center	1.16	[0.44,3.03]	3.42***	[1.65,7.08]
Rural health center	0.60	[0.26,1.41]	0.73	[0.37,1.42]
Government/NGO owned	1.14	[0.32,3.97]	0.83	[0.49,1.41]
Staff HIV training in last year	1.57	[0.67,3.70]	1.74*	[1.05,2.89]
Has nurse-led HIV treatment	0.71	[0.37,1.35]	1.03	[0.58,1.81]
Age of ART program	1.00	[0.83,1.21]	1.01	[0.88,1.15]
Has on-site CD4 analysis	0.68	[0.30,1.54]	0.69	[0.36,1.32]
**Indications of failure in prior 6 months**				
Immunological criteria	0.90	[0.65,1.26]	2.60**	[1.27,5.29]
Clinical criteria	0.77	[0.57,1.04]	1.28	[0.90,1.83]
**CD4 at time of survey**				
*n*	5,162		3,867	
**Patient characteristics**				
Female	1.14*	[1.00,1.30]	1.15	[0.97,1.36]
Age at time of survey	1.01*	[1.00,1.01]	1.01*	[1.00,1.02]
Years on ART	1.04	[0.99,1.10]	0.93	[0.87,1.00]
**Facility characteristics**				
Facility type (ref: Hospital)				
Urban health center	2.15*	[1.13,4.09]	0.86	[0.35,2.13]
Rural health center	0.74	[0.40,1.36]	0.21**	[0.08,0.53]
Government/NGO owned	1.23	[0.51,2.96]	0.93	[0.46,1.90]
Staff HIV training in last year	2.42**	[1.35,4.33]	0.49*	[0.24,0.98]
Has nurse-led HIV treatment	0.78	[0.49,1.22]	2.49*	[1.17,5.30]
Age of ART program	0.92	[0.80,1.05]	1.06	[0.88,1.26]
Has on-site CD4 analysis	1.86*	[1.05,3.31]	1.30	[0.54,3.14]
**Indications of failure in prior 6 months**				
Immunological criteria	1.00	[0.79,1.27]	1.25	[0.83,1.87]
Clinical criteria	0.93	[0.74,1.17]	1.34*	[1.00,1.80]

*p < 0.05

**p < 0.01

*** p < 0.001

## Discussion

Kenya and Uganda have both made tremendous gains in enrolling patients on ART. However, in the setting of increased financial constraints and attention towards quality of care, a careful examination of monitoring practices is essential. As monitoring guidelines for ART patients transition to include viral load testing, our findings raise relevant questions about clinic visit frequency, laboratory capacity, and adherence to international and national norms.

We found that patients were visiting the clinic quite frequently, and on average 90% of established patients had a visit every three months in 2012. Since most of these patients were likely to be stable and required little or no intervention, it is important to consider whether quarterly clinic visits for this established stable population on ART is necessary, as a reduction in visit frequency could have a significant impact on facility costs and efficiency [31]. Reducing ART visit frequency could also lessen the burden on patients, as exit interviews conducted as part of the larger ABCE project found that patients waited and traveled longer for ART services than other types of care [28,29]. A recent analysis from Kenya demonstrated the cost-effectiveness of a model that shifts care from health facilities to the community, thereby reducing the frequency of ART visits [32]. Another study showed that among virally suppressed patients, there was no difference in the probability of continued suppression among patients who returned for care at different frequencies (i.e., three, four, or six months later) [33]. Further research is needed to establish whether refining the frequency of clinic visits yields similar results across ART program settings.

Our study found that most ART patients in our sample regularly visited facilities, suggesting that patient monitoring may be primarily limited by facility resources and actions rather than care-seeking behaviours. While low-tech, point-of-care weight monitoring was feasible and performed according to guidelines at most facilities, less than 50% of patients in care received a CD4 test during the previous six months in both Kenya and Uganda. Given that ART patient volumes dramatically increased during the study period, the overall number of CD4 tests has undoubtedly grown; at the same time, facilities barely kept pace or fell behind in the proportion of their patients receiving CD4 tests for continuous monitoring. In Uganda, we found that facilities where staff HIV training was performed within the prior year the odds of having a CD4 at baseline or afterwards was lower than in facilities where there was no such training. This could potentially be explained by an even more dramatic increase in patient volumes in facilities with additional training/expertise leading to demand for testing outstripping testing capacity. Alternatively, facilities where HIV training was performed may have noted less benefit in recurrent CD4 counts, and reserved testing for patients who appeared clinically ill or had another indication for CD4 testing. We also found that CD4 testing was strongly related to having on-site analysis capacity. As CD4 testing remains one of the primary means for determining ART initiation, our results support equipping facilities to analyze CD4 tests, or at a minimum improving systems through which off-site CD4 test analysis can occur efficiently and at low cost. Our findings complement previous research on the benefits of point-of-care CD4 testing to reduce attrition between testing and initiation [34,35].

Experiences with off-site CD4 analysis are also highly relevant as countries begin scaling up viral load monitoring. While viral load testing offers benefits over immunological and clinical monitoring [8], this guideline shift does not expressly address the underlying issue of access to monitoring. For instance, Uganda plans to use its existing sample transport referral network to deliver and analyze viral load samples at a centralized location in Kampala; while this approach may help with the initial roll-out of viral load testing, it does not improve point-of-care needs and may remain limited by the same infrastructure challenges underlying CD4 testing [36]. New guidelines call for immunological testing in instances where viral load is unavailable [8,20], but our findings demonstrate that such alternatives (i.e., CD4 testing) may remain inaccessible for many patients, particularly in rural areas. Greater policy attention is needed to address these limitations to laboratory capacity in sub-Saharan Africa, especially as the need for more ART services in rural areas is likely to grow. To this end, the development of effective point-of-care viral load testing could be an important innovation [37].

Our findings should be viewed in light of a number of limitations. First, we only examined tests and visits recorded in patient charts. While these data reflect the information available to providers for monitoring purposes, we cannot quantify how much record-keeping practices affected our results concerning gaps between recommended and observed testing practices. Second, patient charts did not contain demographic characteristics, such as educational levels and household wealth, which could be important determinants of health behaviors. Third, chart storage for deceased or defaulted patients may vary across facilities, a practice that could introduce biases. Further, electronic medical records received from six facilities may differ from the paper records used at the remaining facilities. At the two Ugandan facilities with electronic records, 100% of patients received a CD4 test at initiation. Since we sampled only a few facilities with electronic records, we are unable to determine if this relationship was due to record-keeping procedures, overall managerial and financial capacity, or a causal relationship between electronic records and better monitoring.

Despite these limitations, our study identifies a clear gap between prior immunological monitoring guidelines and clinical practice. These findings are particularly relevant as the number of patients eligible for ART continues to increase due to changing initiation guidelines.^5^ Future research will need to address the frequency of routine clinic visits and testing, while national HIV treatment programs will also need to look toward improving current laboratory capacity. Striking this balance in terms of patient visit quantity and quality is critical to ensuring that patients receive the maximum benefits of ART.
